# Exploratory Study of Palliative Care Utilization and Medical Expense for Inpatients at the End-of-Life

**DOI:** 10.3390/ijerph19074263

**Published:** 2022-04-02

**Authors:** Hui-Mei Lin, Chih-Kuang Liu, Yen-Chun Huang, Ming-Chih Chen

**Affiliations:** 1 Taipei City Hospital, RenAi Branch Nursing Supervisor, Taipei 106, Taiwan; b0739@tpech.gov.tw; 2 Graduate Institute of Business Administration, Fu Jen Catholic University, New Taipei City 242, Taiwan; charles.jhs@gmail.com (C.-K.L.); hivicky92@gmail.com (Y.-C.H.); 3 Artificial Intelligence Development Center, Fu Jen Catholic University, New Taipei City 242, Taiwan; 4 Department of Urology, Fu Jen Catholic University Hospital, New Taipei City 242, Taiwan

**Keywords:** palliative care utilization, machine learning methods

## Abstract

Background: Previous research mostly analyzed the utilization of palliative care for patients with cancer, and data regarding non-cancer inpatients are limited. Objectives: This research aimed to investigate the current situation regarding palliative care and the important factors that influence its utilization by inpatients (including inpatients with and without cancer) at the end of their lives. We also explored the feasibility of establishing a prediction model of palliative care utilization for inpatients at the end of their lives. These findings will allow medical staff to monitor and focus on those who may require palliative care, resulting in more end-of-life patients receiving palliative care and thereby reducing medical expense and improving their quality of life. Methods: This was a retrospective study based on real-world health information system (HIS) data from 5 different branches of Taipei City Hospital between 1 January 2018 and 31 December 2018 that enrolled a total of 1668 deceased inpatients. To explore palliative care utilization at the end of life, we used 5-fold cross-validation in four different statistical models to obtain the performance of predictive accuracy: logistic regression (LGR), classification and regression tree (CART), multivariate adaptive regression spline (MARS), and gradient boosting (GB). The important variables that may affect palliative care utilization by inpatients were also identified. Results: The results were as follows: (1) 497 (29.8%) inpatients received palliative care; (2) the average daily hospitalization cost of patients with cancer who received palliative care (NTD 5789 vs. NTD 12,115; *p* ≤ 0.001) and all patients who received palliative care (NTD 91,527 vs. NTD 186,981; *p* = 0.0037) were statistically significantly lower than patients who did not receive palliative care; (3) diagnosis, hospital, and length of stay (LOS) may affect palliative care utilization of inpatient; diagnosis, hospitalization unit, and length of hospitalization were statistically significant by LGR; (4) 51.5% of patients utilized palliative consultation services, and 48.5% utilized palliative care units; and (5) MARS had the most consistent results; its accuracy was 0.751, and the main predictors of palliative care utilization are hospital, medical expense, LOS, diagnosis, and Palliative Care Screening Tool-Taiwan version (TW-PCST) scores. Conclusions: The results reveal that palliative care utilization by inpatients remains low, and it is necessary to educate patients without cancer of the benefits and advantages of palliative care. Although data were limited, the predictability of the MARS model was 0.751; a better prediction model with more data is necessary for further research. Precisely predicting the need for palliative care may encourage patients and their family members to consider palliative care, which may balance both physical and mental care. Therefore, unnecessary medical care can be avoided and limited medical resources can be allocated to more patients in need.

## 1. Introduction

According to the World Health Organization, palliative care is defined as “an approach that improves the quality of life of patients and their families facing problems associated with life-threatening illnesses through the prevention and relief of suffering by employing early identification and impeccable assessment and treatment of pain and physical, psychosocial, and spiritual problems.” [1]. A patient with a severe disease that receives poor-quality medical care and service, such as untreated symptoms and unmet psychosocial and personal care needs, is a burden for the caregiver and results in low patient and family satisfaction [2]. Palliative care focuses on patient-centered symptomatic treatment, pain management, and hospice care, and its ultimate goal is to achieve the best possible quality of life for patients, their family, and their caregivers [3]. Most inpatients die in the hospital, and in a clinical aspect, seriously ill patients are often transferred between ICUs and standard rooms during later stages of their disease. In addition, they often have to decide whether to continue treatment or instead receive palliative care. The benefit for inpatients receiving early proactive palliative care includes a shorter hospital stay [4]. However, studies have shown that palliative care utilization by inpatients remains low and dying patients rarely receive palliative care [5].

The principle of palliative care can also be applied during the early stages of any serious illness. If the illness has the potential to be cured, palliative care can and should be provided to the patient while simultaneously providing treatment [6]. The value of palliative care lies in its ability to raise the quality of life and lower the cost of treatment. In addition, palliative care services also reduce the mean hospitalization duration and cost [5,7,8]. It has also been suggested by the American Society of Clinical Oncology and the Institute of Medicine that patients should not receive intensive treatment at the end of their lives [7]. However, many patients with late-stage cancer still undergo intensive treatments up to a month before their death [8]. These increase the healthcare costs not only of patients but also of the medical system. Palliative care services have become standard end-of-life care for critically and terminally ill patients. In addition to providing the best possible quality of life and emphasizing the dignity of life, it also contributes to the improvement of the quality of medical care.

Taiwan was the first Asian country to enact palliative care legislation. In 2012, the Taiwan National Health Insurance Administration introduced a family palliative care consultation fee to encourage physician teams to communicate with families of terminally ill patients and discuss “do not resuscitate” (DNR) directives and palliative care. Within 24 h of a patient’s admission, nursing staff of Taipei City Hospital use the patient’s Palliative Care Screening Tool-Taiwan version (TW-PCST) score to identify whether family palliative care consultation is required. The TW-PCST evaluates four categories: (1) the severity of the baseline disease process; (2) the severity of the comorbidity process; (3) functional performance status; and (4) whether the patient has had frequent admissions or intensive care unit (ICU) stays [9].

Palliative care is focused on improving a patient’s quality of life, and it may be administered to patients with severe illnesses at different stages of the disease [10]. Hospital-based palliative care services include palliative consultation services and care units. Palliative consultation services include an interdisciplinary team composed of physicians (including generalists, specialists, and psychiatrists), nurse practitioners, registered nurses, social workers, psychologists, chaplains, pharmacists, and volunteers. The goal of consultation is to support the referring clinicians; usually, the consultation team provides suggestions to the attending doctor. The palliative care unit can either be operated by an attending doctor responsible for the patient or apply the model of consultation. Patients who are transferred to the palliative care unit are those with symptoms that are difficult to control and medical requirements that cannot be fulfilled in other settings. Families that need a higher level of support, patients who need to transfer out of the ICU, and patients who are on the verge of dying are usually transferred to the palliative care unit. Palliative care provides the needs of not only the patients but also of their family members, avoiding unnecessary treatment and critical care of terminally ill patients [11,12,13,14]. Palliative care can not only be administered to patients with cancer but also to non-cancer inpatients at the end of their lives. However, previous research has mostly analyzed the application of palliative care to patients with cancer, and data regarding its administration to non-cancer patients are limited. Additionally, the methods of previous studies mostly relied on questionnaires or system data to analyze related factors. In contrast, this study utilized the health information system (HIS) data of hospitals, establishing a machine learning model via real-world data to predict factors that contribute to palliative care administration to inpatients.

Most previous research used the basic statistical method of logistic regression (LGR) to analyze the factors influencing palliative care use. In recent years, many researchers used different machine leaning techniques to enhance prediction models. Huang et al. found that XGBoost and MARS both had the higher performance of survival prediction in elderly CABG patients. RF was the best model in early glaucoma detection [15,16]. Due to the accuracy of LGR model in our research being under the average performance compared to machine learning methods, we tried some machine learning algorithms for higher accuracy. Therefore, this study not only used the traditional LGR method but also applied different machine learning methods to make prediction models with real-world data. Finally, the comparison of the models with several predictive performances is provided.

The goal is to establish a prediction model that can identify who among the inpatients will eventually require palliative care. The application of this model can help medical staff in predicting patients likely to receive palliative care, which will improve the efficiency of medical services distribution and allocation and prevent unnecessary medical treatment while simultaneously increasing the quality of the lives of terminally and seriously ill patients.

## 2. Materials and Methods

### 2.1. Study Design and Population

**Figure 1:**
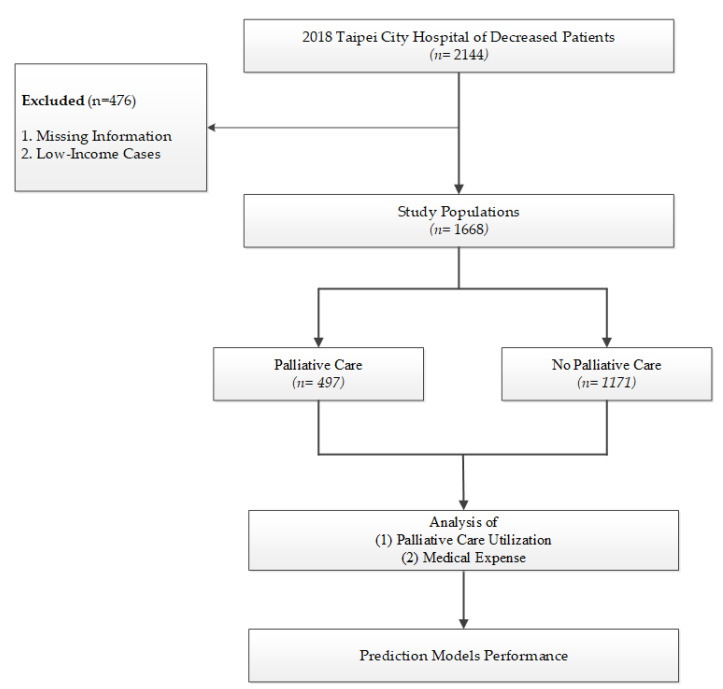
Research flow chart of patients deceased between 1 January 2018 and 31 December 2018.

This is a retrospective study that used real-world HIS data from Taipei City Hospitals. The study population were deceased patients who were ≥20 years old between 1 January 2018 and 31 December 2018. This study excluded missing information (*n* = 11) and low-income cases (*n* = 476). The social welfare policy in Taiwan provides low-income households with a reduction or exemption of medical expenses. Because the medical expenses were analyzed to compare the differences in palliative care use, low-income cases were excluded to avoid underestimation. A total of 1668 deceased inpatients were enrolled in this study (Figure 1).

### 2.2. Baseline Variables

The following patient data were collected and analyzed: (1) sex (female/male); (2) age (<65, 65–79, and ≥80 years); (3) advanced directives (whether the patient had DNR con-sent); (4) diagnosis (cancer or non-cancer); (4) hospital (Zhongxing, Heping Fuyou, and Yangming branches did not have a palliative care unit); (5) family palliative care consultation; (6) palliative consultation services; (7) admission to palliative care units; (8) LOS; (9) TW-PCST score; and (10) medical expenses.

### 2.3. Statistical Analysis

The Chi-square test was used for categorical variables, and the results were expressed as *N* (%). The *t*-test was used for continuous variables, and the results were expressed as mean ± standard deviations (std). Moreover, this research used R statistical software (Version 3.4.3; The R Foundation, Vienna, Austria).to predicted results by using different machine learning methods.

Logistic regression is a basic prediction methodology for predicting classification problem (0) and (1). Classification and regression-tree algorithm (CART) uses Gini index to calculate the impurity in each attribute segmentation until the decision tree is constructed. MARS is a flexible regression and it can handle multiple complex data [17]. The technique of gradient boosting (GB) based is added recursively and adjust the residuals, the main goal in this model is increased the computational speed [17,18,19]. All machine learning methods were applied in R software version 3.6.2 and the package was as follows: glm package for LGR; rpart package for CART; earth package for MARS; and gbm package for GB. The statistical analyses were two-sided, and a *p*-value < 0.05 was considered statistically significant.

## 3. Results

### 3.1. Study Population Characteristics

The baseline characteristics of the 1668 patients in this study are shown in Table 1. Among the patients, 54.92% were male, and 60.13% were >80 years old. In addition, 79.86% had DNR consent, 79.08% were patients without cancer. Most patients were ad-mitted at Renai (34.17%) and Heping Fuyou (20.80%) hospitals. Of the patients, 73.50% participated in family palliative care consultation, and 77.40% and 85.55% did not utilize palliative consultation services and palliative care units, respectively. Palliative care-de-noted patients used palliative consultation services or palliative care or both. The per-centage of overall use of palliative care was 29.80%. The average LOS was 27.3 days; however, 46.64% were hospitalized for 1–10 days. The average TW-PCST score was 3.51 points, and the average medical expense was NTD 194,818.

### 3.2. Palliative Care Utilization for Inpatients

Based on palliative care utilization, we divided the patients into those who utilized palliative care and those who did not. The number of patients with and without cancer (cancer, 33.00% vs. 15.80%; non-cancer, 67.00% vs. 84.20; *p* ≤ 0.001 ***), hospital (*p* ≤ 0.001 ***), LOS (*p* ≤ 0.001 ***), and TW-PCST scores (*p* ≤ 0.001 ***) were statistically significantly different between those who did and did not utilize palliative care. The results are summarized in Table 2.

### 3.3. Impact of Palliative Utilization on Medical Expense

As shown in Table 3, the average medical expense of daily hospitalization of all patients (NTD 5789 vs. NTD 12,115; *p* ≤ 0.001 ***) and patients with cancer who received palliative care (NTD 91,527 vs. NTD 186,981; *p* = 0.0037 **) were statistically significantly lower than patients who did not receive palliative care.

### 3.4. Palliative Care Utilization

Palliative care alleviates psychological and physical pain and eventually fulfills the patient’s last wishes at the end of their life. Renai and Zhongxiao hospitals have built hospices intended for palliative care. Table 4 shows that palliative care can still be administered even in hospitals that do not have palliative care units.

### 3.5. Prediction Model for Palliative Care Utilization

This study used logistic regression analysis to evaluate the significant factors in palliative care utilization. Table 5 shows variables of hospitals (*p* = 0.01866 *), medical expenses (*p* < 0.001 ***), and TW-PCST scores (*p* ≤ 0.001 ***) that were all statistically significant. The accuracy of LGR was not good enough; thus, we added three machine learning prediction models, namely, CART, MARS, and GB, as shown in Table 6. The rate of palliative consultation services accuracy was between 0.6736 and 0.7510. MARS had the highest accuracy, followed by GB.

Table 6 shows four different machine learning models, namely, LGR, CART, MARS, and GB. The rate of accuracy was between 0.6736 and 0.7510. MARS had the highest accuracy followed by GB.

## 4. Discussion

### 4.1. Factors Influencing Palliative Care Utilization

In this study, the utilization rate of palliative care for inpatients at the end of their life was 29.8% (497/1668), which was higher than that of inpatients with liver and kidney syndrome in other studies (13.4%) [5]. In this study, only 298 (20.84%) people aged >80 years used palliative care resources, which is relatively low. In the future, the utilization rate of palliative care can be promoted for the elderly to achieve a good death.

The factors for palliative care utilization include the diagnosis of cancer/non-cancer, hospital they were admitted in, and the duration of hospitalization. Additionally, we discovered that palliative care utilization of patients with cancer (46.9%, 164/349) was higher than that of patients without cancer (25.24%, 333/1319). Patients without cancer who are terminally ill must also be encouraged to consider palliative care. A previous study that used questionnaires found that cultural and religious factors heavily impacted whether patients should receive palliative care [18]. This was mainly because discussing oncoming death is a sensitive issue and considered inappropriate. In this study, the use of prediction models and analysis of data from the medical database revealed that the important factors that affect palliative care utilization at the end of life are the hospital patients were admitted in, medical expense, LOS, diagnosis, and TW-PCST scores. Understanding the low utilization of palliative care by inpatients is associated with a decrease in the usage of palliative care [3,5]. Consideration of palliative care usually occurs during the late stages of an illness; however, some terminally ill patients are still receive treated aggressively, leading to resources for palliative care being used insufficiently. Therefore, an early integrated and collaborative practice is necessary to face the challenges at the end of life [20]. Encouraging end-of-life discussions with patients and their family members increases not only patient satisfaction but also palliative care utilization [3,21,22].

### 4.2. Effect of Usage of Palliative Care on Medical Expense

In terms of the average daily medical expense, this study found that patients who received palliative care had lower medical expense than those who did not. P. May suggested that patients who received palliative care during hospitalization had significantly lower medical expense regardless of the diagnosis, and the difference of patients with cancer or patients with multiple comorbidities [19]. Moreover, Meyer et al. revealed that palliative care reduced the average medical expense and duration of hospitalization [3,7,8]. The results of this study are consistent with those in the literature; therefore, palliative care for inpatients reduces medical expenses.

### 4.3. Decision of Palliative Care Models

Meanwhile, palliative care consultation remains low [23,24], and more than half of the inpatients did not undergo palliative care consultations [21]. As palliative care units are more expensive and require more resources, some hospitals do not have these units. Our study found that in the model of palliative care, more than half adopt the model of palliative shared care, which allows the administration of palliative care despite the lack of palliative care units. The literature suggests that the ideal set-up is where a large hospital adopts both models [24], wherein a specific department administers palliative care and proficient interdisciplinary staff addresses serious illnesses as demanded by family members. On the other hand, a consultation team with ample resources would be able to provide adequate care to patients and their families while sharing the importance of palliative care. By promoting and encouraging palliative shared care, palliative care utilization can increase. As many patients die in hospitals, those who are terminally ill must receive palliative care if required to ensure proper end-of-life care, regardless of whether they are in palliative care units or in general wards. Palliative shared care can overcome the limitations of location and concept of the institution without palliative care units; referring the patients in a timely manner when the original medical team discovers the requirement of patients or family members physically, mentally, or socially. This practice not only enables the medical team to provide optimal physical and mental care to the patients and their family members but also increases the experience and understanding of palliative care to improve the overall quality of care.

## 5. Conclusions

The results of our study revealed that palliative care utilization by inpatients is low. However, several factors may promote and increase utilization by patients without cancer at the end of their lives. The predictability of the MARS model is 0.751, which can be used to predict the patients who will require palliative care, ultimately resulting in reduced medical expenses and increased quality of life in terminally ill patients.

## 6. Limitation

Limitations of this study include the following: (1) low-income cases were excluded to avoid underestimation of medical expenses, and (2) this study only analyzed data from the Taipei City Hospital. The utilization of palliative care resources by inpatients in a municipal public hospital at the end of life was presented and we tried to build a preliminary prediction model. It is suggested that a study with a larger dataset from multiple hospitals can be conducted in the future.

## Figures and Tables

**Table 1:** Baseline characteristics.

Baseline		*N* %	(%)
Overall population		1668	100%
Sex	Male	916	54.92%
	Female	752	45.08%
Age group	<65	225	13.49%
	65–79	440	26.38%
	≥80	1003	60.13%
Age (mean, std)		80.32 (13.49)	
DNR	Yes	1332	79.86%
	No	336	20.14%
Diagnosis	Cancer	349	20.92%
	Others	1319	79.08%
Hospital	Zhongxiao	287	17.21%
	Zhongxing	243	14.57%
	Heping Fuyou	347	20.80%
	Yangming	221	13.25%
	Renai	570	34.17%
Family palliative care consultation	Yes	1226	73.50%
	No	442	26.50%
Palliative consultation services	Yes	377	22.60%
	No	1291	77.40%
Palliative care units	Yes	241	14.45%
	No	1427	85.55%
Palliative care ^a^	Yes	497	29.80%
	No	1171	70.20%
Length of stay (days)	1–10	778	46.64%
	11–20	348	20.86%
	21–30	219	13.13%
	>30	323	19.36%
Length of stay (mean, std)		27.30 (73.63)	
TW-PCST score	(Mean, std)	3.51 (2.16)	
	Unknown (*N*)	378	
Medical expense, NTD (mean, std)		194,818 (426,945)	

TW-PCST scores: Taiwanese version-palliative care screening tool scores; LOS: length of stay; ^a^: The patients used either palliative consultation services or palliative care or both.

**Table 2:** Inpatients at end of life with and without palliative care utilization.

Baseline		Palliative Care		No Palliative Care		*p*
		(*n* = 497)		(*n* = 1171)		
		*N*	%	*N*	%	
Sex	Male	237	47.69	515	43.98	0.1641
	Female	260	52.31	656	56.02	
Age group	<65	61	12.27	164	14.01	0.5232
	65–79	138	27.77	302	25.79	
	≥80	298	59.96	705	60.20	
Age mean (std)		80.91 (13.12)		80.07 (13.64)		0.2453
DNR	Yes	406	81.69	926	79.08	0.2237
	No	91	18.31	245	20.92	
Diagnosis	Cancer	164	33.00	185	15.80	≤0.001
	Non-cancer	333	67.00	986	84.20	
Hospital	Zhongxiao	146	29.38	141	12.04	≤0.001
	Zhongxing	86	17.30	157	13.41	
	Heping Fuyou	38	7.65	309	26.39	
	Yangming	7	1.41	214	18.27	
	Renai	220	44.27	350	29.89	
Family palliative care consultation	Yes	128	25.75	314	26.81	0.6536
	No	369	74.25	857	73.19	
Length of stay (days)	1–10	185	37.22	593	50.64	≤0.001
	11–20	126	25.35	222	18.96	
	21–30	80	16.10	139	11.87	
	>30	106	21.33	217	18.53	
TW-PCST score	(Mean, std)	3.54 (2.13)		3.50 (2.17)		0.7459
	Unknown (*N*)	143	28.77	253	21.61	0.0312

TW-PCST scores: Taiwanese version-palliative care screening tool scores; LOS: length of stay; SD: standard deviation.

**Table 3:** Medical costs with and without palliative care.

Baseline		Palliative Care	No Palliative Care	*p*
		Medical Cost, NTD	Medical Cost, NTD	
		(*n* = 497)	(*n* = 1171)	
Sex	Male	195,004 (562,629)	214,813 (445,634)	0.6117
	Female	154,594 (316,466)	187,767 (362,371)	0.2031
Age group	<65	176,236 (384,486)	193,772 (301,040)	0.7206
	65–79	222,033 (680,109)	209,985 (442,960)	0.8492
	≥80	154,191 (333,399)	202,019 (419,551)	0.0557
DNR	Yes	159,637 (320,538)	192,234 (356,897)	0.0995
	No	247,553 (840,891)	243,300 (570,642)	0.9645
Diagnosis	Cancer	91,527 (107,970)	186,981 (427,035)	0.0037
	Non-cancer	217,206 (554,796)	205,908 (408,255)	0.7328
Family palliative care consultation	Yes	197,256 (471,275)	206,306 (344,590)	0.7396
	No	113,692 (429,506)	193,673 (554,107)	0.105
Length of stay mean (std)		30.33 (81.60)	26.02 (69.97)	0.3046
TW-PCST score	(Mean, std)	158,662 (331,716)	189,907 (360,096)	0.1557
	Unknown	254,741 (37,115)	217,887 (684,968)	0.5738
Medical expense (std)		175,734 (461,906)	202,918 (411,150)	0.2567
Average (Medical expense)/average (LOS)		5789.1 (3855.4)	12,115.8 (13,991.5)	≤ 0.001

**Table 4:** Percentage utilization of two types of palliative care services.

Hospital	Palliative Consultation Services (*N* = 256)		Palliative Care Units (*N* = 241)	
	*N*	%	*N*	%
Renai	79	30.86	141	55.83
Zhongxiao	46	17.97	100	44.17
Zhongxing	86	33.59	–	
Heping Fuyou	38	14.84	–	
Yangming	7	2.73	–	

–: No palliative care units in the hospital.

**Table 5:** The value of coefficient by logistic regression model.

Baseline	Estimate	Error	Pr (>Chi)
Intercept	−1.660	0.4372	
Hospital	−0.2351	0.04925	0.01866 *
Sex	0.130	0.1251	0.18719
Age	0.0059	0.0046	0.15969
DNR	0.2419	0.1618	0.18063
Medical expense	−3.080 × 10^−6^	6.398 × 10^−7^	<0.001 ***
Family palliative care consultation	0.1030	0.1443	0.87391
TW-PCST scores	0.09851	0.01695	<0.001 ***
LOS (days)	0.01741	0.00351	0.24705

TW-PCST scores: Taiwanese version-palliative care screening tool scores; LOS: length of stay; *: *p* < 0.05; ***: *p* < 0.001.

**Table 6:** Performance evaluation of prediction models.

	Accuracy	Kappa	Sensitivity	Specificity	AUC
LGR	0.6736	0.2673	0.8012	0.4625	0.7058
CART	0.6943	0.3495	0.8464	0.5213	0.7286
MARS	0.7510	0.4501	0.8692	0.5722	0.7847
GB	0.7357	0.4311	0.8865	0.5251	0.8213

LGR: logistic regression; CART: classification and regression tree; MARS: multivariate adaptive regression splines; GB: gradient boosting.

## Data Availability

The data presented in this study are not available on request from the corresponding author. Due to the General Data Protection Regulation, the data presented in this research are not publicly available.
